# How much trachomatous trichiasis is there? A guide to calculating district-level estimates

**Published:** 2019-02-10

**Authors:** Anthony W Solomon, Assumpta Lucienne Françoise Bella, Nebiyu Negussu, Rebecca Willis, Hugh R Taylor

**Affiliations:** 1 Medical Officer for Trachoma: Department of Control of Neglected Tropical Diseases, World Health Organization, Geneva, Switzerland.; 2 Professeur: Faculté de Médecine et des Sciences Biomédicales, Université de Yaoundé I, Yaoundé, Cameroun.; 3 Neglected Tropical Diseases Team Leader: Federal Ministry of Health, Addis Ababa, Ethiopia.; 4 Data and Analytics Team Manager: International Trachoma Initiative, Task Force for Global Health, Decatur GA, USA.; 5 Harold Mitchell Professor of Indigenous Eye Health: University of Melbourne, Melbourne, Australia.

**Estimating the number of people with trachomatous trichiasis allows managers to plan surgical services and obtain the resources needed to eliminate this painful condition.**

**Figure:**
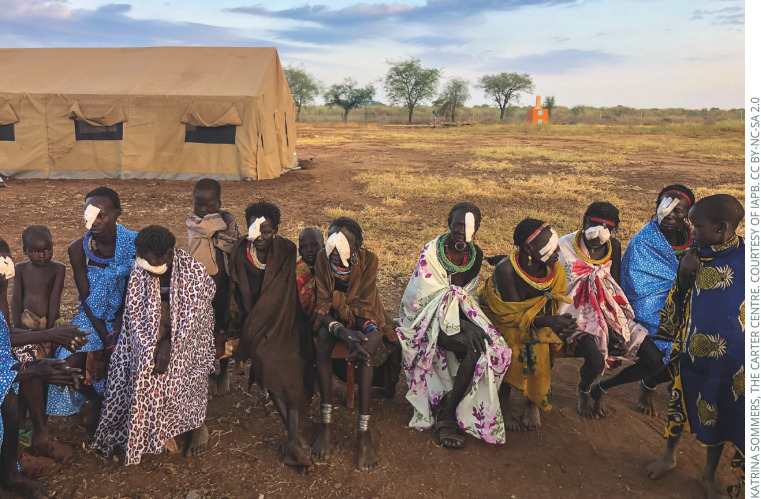
Patients who have undergone trachomatous trichiasis surgery. **SOUTH SUDAN**

In some people living in trachoma-endemic areas, repeated conjunctival *Chlamydia trachomatis* infection eventually leads to trachomatous trichiasis (TT), in which one or more eyelashes are misdirected so that they rub on the eyeball.^1^^,^^2^ Untreated, this can scar the cornea and result in permanent blindness. Estimating the likely number of people with TT helps programme managers to plan surgical services and secure resources.^3^^,^^4^ Recent work to complete high-quality baseline mapping of suspected trachoma- endemic populations worldwide^5^^,^^6^ and to standardise the systems and methodologies of trachoma impact surveys, pre-validation surveillance surveys, and TT-only surveys^7^^-^^10^ has facilitated production of more accurate estimates of the number of people with TT.^11^^,^^12^

This paper, intended for programme managers and their supporters, explains the concepts involved in estimating TT prevalence and discusses programmatic applications of that information. We hope it will help these stakeholders to use their TT data with greater confidence.

## 1 What does *prevalence* mean?

The *prevalence* of a disease is the percentage of people in a defined population who are affected by that disease at a particular time. In this definition, it is important to clearly identify the *defined population.* The prevalence of TT can be estimated for a single community,^13^ a single administrative division of a country,^14^ or a whole country.^15^ At each of these levels, prevalence could be estimated in (for example) people of all ages,^16^ people aged ≥15 years,^17^ women aged ≥15 years,^18^ or some other subset of the population, such as indigenous inhabitants,^19^ so it is important to be clear on the group being studied.

## 2 At what administrative level are TT prevalence estimates usually generated?

For trachoma elimination purposes, the World Health Organization (WHO) defines districts as “the normal administrative unit for health care management,” which “for purposes of clarification, consists of a population unit between 100,000 and 250,000 persons”.^20^ Although surveys at baseline may be conducted at larger-than-district-level in order to start the programme,^20^^-^^23^ it is currently recommended that impact, pre-validation surveillance and TT-only surveys are done at district level. In the real world, the term *district* has different meanings in different contexts. Because of this, and because the administrative level surveyed may change from one round of surveys to the next, we will subsequently refer here instead to *evaluation units* (EUs), a generic term for the population unit surveyed, regardless of size – even though we used ‘district-level’ (for readability) in the title of this article.

Regardless of the administrative level at which EUs are framed, data should be interpreted and applied at that same level; in other words, local data should inform local action.

## 3 What data are used to generate TT prevalence estimates?

To estimate TT prevalence, population-based surveys are recommended.^24^ Standard trachoma baseline, impact, pre-validation surveillance and TT-only surveys are all population-based surveys. These all employ sampling, in which a small proportion of EU residents are selected for examination, using a random or quasi- random sampling technique, with data on individuals examined considered to be representative of the EU population overall.

In trachoma surveys, the sampling strategy used is often two-stage cluster sampling.^24^ The first stage involves selecting 20-30 communities *(first-stage clusters)* from the set of all communities in the EU. The second stage, undertaken within each selected community, involves selecting a fixed number of households *(second-stage clusters,* often grouped within a single compact segment^25^^,^^26^) from the set of all households in the community. (In *compact segment* sampling, a sketch map is drawn of the sampled first-stage cluster, and the area then divided into sub-clusters or segments containing approximately equal numbers of households. One segment is selected by random draw.) All qualifying individuals living in selected households are asked to participate (in compact segment sampling, all residents of all households in the compact segment are asked to participate^27^), and both eyes of consenting individuals are examined by certified trachoma graders.^5^^,^^10^

A *person with trichiasis* is defined as someone in whom, in at least one eye, one or more eyelashes touch the eyeball or there is evidence of recent removal of in-turned eyelashes. Although determining whether eyelashes that have been removed were in-turned is difficult, this may be very important: in Fiji, for example, many adults practice eyelash epilation in the absence of trichiasis.^28^

## 4 How are survey data processed to generate a TT prevalence estimate?

Before any calculations are performed, data are screened for possible errors, such as missing data from some included communities, inclusion of data from communities lying outside the EU, or missing data from particular demographic subsets. Best practice^29^ calls for data to be cleaned and analysed by an objective data manager who works in collaboration with, but at arm's length from, the health ministry, employing standardised methods. Outputs are checked and approved by the responsible health ministry.^5^^,^^6^ Then if, for example, 2,000 people aged ≥15 years living in the EU were examined, and 10 of them had TT, the raw TT prevalence in ≥15-year-olds would be 0.5%. (In this example, the calculation is: Prevalence = (10/2,000) × 100 = 0.5%. The number 10 here is referred to as the numerator, and 2,000 is the denominator.)

Such an estimate may not represent the true EU-level prevalence in ≥15-year-olds, for two reasons. First, it is rare that everyone resident in selected households is examined: women and older adults are both more likely to be examined in house-to-house surveys, and more likely to have trichiasis, than men and younger adults, respectively. Second, the number of ≥15-year-olds examined in a set number of households (say, n households) in each community varies. Communities in which a greater number of ≥15-year-olds are examined should not contribute more weight to the EU-level prevalence.

To compensate, partially, for the first problem (unbalanced recruitment of different age and gender groups), standard trachoma survey analyses adjust the first-stage cluster data by gender and age in five-year age bands. This can be conceptualised as filling in missing data from individuals who were resident in selected households but not examined, using the assumption that their risk of trichiasis was similar to that of residents of similar age and gender who were examined. To compensate for the second problem (varying numbers of individuals examined per cluster), the age- and gender-adjusted first-stage cluster-level TT percentages are averaged to generate the EU-level TT prevalence. This gives equal weight to each of the firststage clusters, as if the same number of ≥15-year-olds had been examined in each one.

## 5 How accurate are TT prevalence estimates generated from cluster- sampled surveys?

Estimates generated through sampling are subject to two types of error: bias and chance. Bias is present if the people included in the sample are systematically different to the EU population as a whole: under- representation of adults with jobs that result in absence on the day of the survey, for example. Gender- and age-adjustment, as described above, attempts to partially correct for this problem, but cannot fully compensate for it. (Biases cannot be quantified, and no amount of statistical manipulation should be considered to completely remove their effect.)

Chance affects prevalence estimates through sampling variation: if a different sample of 2000 ≥15-year-olds living in the EU had been examined, a different prevalence estimate might have been generated. The chance-induced uncertainty of an estimate produced through sampling can be quantified: it is expressed as a confidence interval. A 95% confidence interval suggests that, based on the observed data, if surveys using the same methodology were repeated multiple times in the EU, in 95% of instances the prevalence estimate would fall between the confidence interval's lower and upper bounds. Other factors being equal, larger sample sizes will produce narrower confidence intervals.

## 6 Can data from house-to-house case searches be used to generate a TT prevalence estimate instead?

In some programmes, TT case-finding is undertaken through house-to-house searches.^30^ If a very high proportion of households in a very high proportion of the EU's communities are visited, with examination undertaken by appropriately trained examiners, such an exercise could provide a better estimate of TT prevalence than a cluster-sampled survey. (Thinking statistically: by trying to examine everyone, chance is removed, though it is possible that bias is not.)

## 7 What are *known* and *unknown* cases of TT, and why is the distinction important?

The trichiasis prevalence threshold for “elimination of trachoma as a public health problem” is a prevalence of TT unknown to the health system in ≥15-year-olds of <0.2%.^31^ Known cases are people with trichiasis in eyes that have already had surgery for trichiasis, for which surgery has been refused, or for which a surgical date has been agreed. (An aide-memoire for this is: “recurrences, refusals and those already referred”.) In standard trachoma surveys,^10^^,^^32^ when an eye is diagnosed as having trichiasis, the subject is asked if a health worker has ever recommended surgery or epilation for that eye.^33^ This allows accurate determination of the numerator for estimating the prevalence of TT unknown to the health system, as included in Tropical Data's expanded trichiasis report **(http://tropicaldata.knowledgeowl.com/help/demo-project—expanded-trichiasis-report)**.

## 8 What is *postoperative* TT, and why is this important?

Even when surgeons are highly skilled, by 12 months after surgery, at least 8-10% of patients again have TT.^34^^,^^35^ Some of this postoperative TT may be due to under-correction and some to further progression of the underlying scarring processes; the term postoperative TT avoids the need to blame or absolve the surgeon, by simply noting that TT is present after an operation has been performed.^36^ Postoperative TT is probably not optimally managed by repeating the same procedure that was used to treat primary TT, and should be managed by the most experienced trichiasis surgeon or eye specialist available.^36^^,^^37^ All programmes should have a plan for managing postoperative TT, so the expanded trichiasis report includes specific information to assist.

It's important to note that standard trachoma surveys do not provide information on how often TT surgery is successful, because individuals who have been successfully managed will not be recorded as being any different to those who have never had TT.

## 9 Why does the expanded trichiasis report also provide data on trichiasis + TS (trichiasis plus trachomatous scarring)?

Not all trichiasis is caused by trachoma.^38^ The global trachoma programme is currently trying to better understand how to distinguish trachomatous from non-trachomatous trichiasis. As part of this effort, when an eye is diagnosed as having trichiasis, standard trachoma survey systems prompt the examiner to assess the conjunctiva of that eye for the presence or absence of trachomatous scarring (TS); when the eyelid cannot be everted, the eye is presumed to have TS.^6^ Generation of these data was recommended by the 2nd Global Scientific Meeting on TT.^36^

## 10 How does the TT prevalence estimate relate to the number of people who need surgery?

TT prevalence is useful at EU level for determining whether the TT prevalence criterion for elimination as a public health problem has been reached; if it has not, public health-level TT surgery services, including active case finding, are recommended. For service planning, the number of prevalent cases should be determined by multiplying the TT prevalence in ≥15-year-olds by the number of resident ≥15-year-olds in the EU. It is important to remember that the number of prevalent cases is just an estimate. Programmes should aim to cover the entire EU with case finding and TT management; this may identify considerably more or considerably fewer people with TT than indicated by the estimate.

## 11 How is the presence of TT in both eyes accounted for?

In an individual with TT, one or both eyes may need management. When planning surgical services, a requirement to operate on two eyes rather than one increases (in varying proportions) requirements for selected consumables and operating theatre time. The expanded trichiasis report uses data on the proportion of survey subjects who had bilateral disease to provide an estimate of the number of eyes, as well as the number of people, with TT.

## 12 Should my estimate of the number of prevalent cases take into account the number of people managed for TT since the most recent prevalence survey?

No: not unless those people were managed within a few weeks of the survey and no further time has passed. As months and years elapse after a survey, new (incident) cases of TT develop; determining that the TT prevalence is below the TT prevalence threshold for elimination of trachoma as a public health problem^31^ almost always requires a formal prevalence estimate. It's also critical to remember that after the TT prevalence criterion for elimination^31^ has been achieved, the need to provide surgical services does not disappear: programmes should expect incident cases to continue to occur for many years. This is the rationale for the inclusion of “written evidence that the health system is able to identify and manage incident TT cases, using defined strategies, with evidence of appropriate financial resources to implement those strategies”^20^ as a criterion for trachoma elimination.^31^

## 13 Why aren't we talking about the TT backlog or the ultimate intervention goal?

Each of these terms has been used in different ways by different stakeholders, such that their usefulness as labels has been completely eroded. We do not recommend that these terms be used.

## 14 Can we eliminate TT by 2020?

Yes. Unlike active trachoma – where the dynamics of *C. trachomatis* transmission^39^^,^^40^ are fundamental to programme impact, and accelerated intervention, such as biannual antibiotic mass drug administration, has not been shown to make a programmatically significant difference^41^ – the rate of decline in TT prevalence is determined by the resources invested. More systematic TT case-finding – plus good access to trained, certified, resourced, and appropriately motivated surgeons and surgical teams – will lead to faster reductions in the numbers of prevalent cases. That is not to say that speed of service delivery is the only important consideration: quality^42^^,^^43^ is also paramount.

Vision loss from TT is avoidable. Together, we must do everything we can to consign it to history.

The authors alone are responsible for the views expressed in this article and they do not necessarily represent the views, decisions or policies of the institutions with which they are affiliated
